# Electrochemical Probing of Human Liver Subcellular S9 Fractions for Drug Metabolite Synthesis

**DOI:** 10.3390/metabo14080429

**Published:** 2024-08-03

**Authors:** Daphne Medina, Bhavana Omanakuttan, Ricky Nguyen, Eman Alwarsh, Charuksha Walgama

**Affiliations:** Department of Physical & Applied Sciences, University of Houston-Clear Lake, 2700 Bay Area Boulevard, Houston, TX 77058, USA

**Keywords:** human liver S9 fraction, microsomes, biofilm, electrocatalysis, drug metabolites, CYP450, CYP reductase

## Abstract

Human liver subcellular fractions, including liver microsomes (HLM), liver cytosol fractions, and S9 fractions, are extensively utilized in in vitro assays to predict liver metabolism. The S9 fractions are supernatants of human liver homogenates that contain both microsomes and cytosol, which include most cytochrome P450 (CYP) enzymes and soluble phase II enzymes such as glucuronosyltransferases and sulfotransferases. This study reports on the direct electrochemistry and biocatalytic features of redox-active enzymes in S9 fractions for the first time. We investigated the electrochemical properties of S9 films by immobilizing them onto a high-purity graphite (HPG) electrode and performing cyclic voltammetry under anaerobic (Ar-saturated) and aerobic (O_2_-saturated) conditions. The heterogeneous electron transfer rate between the S9 film and the HPG electrode was found to be 14 ± 3 s^−1^, with a formal potential of −0.451 V vs. Ag/AgCl reference electrode, which confirmed the electrochemical activation of the FAD/FMN cofactor containing CYP450-reductase (CPR) as the electron receiver from the electrode. The S9 films have also demonstrated catalytic oxygen reduction under aerobic conditions, identical to HLM films attached to similar electrodes. Additionally, we investigated CYP activity in the S9 biofilm for phase I metabolism using diclofenac hydroxylation as a probe reaction and identified metabolic products using liquid chromatography–mass spectrometry (LC-MS). Investigating the feasibility of utilizing liver S9 fractions in such electrochemical assays offers significant advantages for pharmacological and toxicological evaluations of new drugs in development while providing valuable insights for the development of efficient biosensor and bioreactor platforms.

## 1. Introduction

Pharmaceutical drug discovery is a costly and time-consuming process, involving the screening of millions of molecules to find a single effective and safe drug [1]. The validation of a drug’s efficacy typically relies on a series of in vitro and in vivo studies. Given the resource-intensive and lengthy nature of in vivo research, advancements in in vitro assays that reduce reliance on animal testing are increasingly attractive. In particular, in vitro ADMET (absorption, distribution, metabolism, excretion, and toxicology) processes are crucial for improving predictions of human clinical outcomes. Among these five processes, understanding the metabolic pathways of a drug, including its potential to form toxic metabolites or exhibit altered efficacy, is essential [2,3] and such metabolism studies and pharmacokinetic evaluations provide a comprehensive assessment of a drug’s safety profile [4].

Drug metabolism primarily involves phase I oxidation reactions facilitated by cytochrome P450 monooxygenases (CYP) and phase II conjugation reactions carried out by UDP-glucuronosyltransferases and sulfotransferases, which help in drug detoxification and excretion [5,6]. In vitro systems utilize subcellular fractions, tissues, cells, and enzymes derived from the human liver to investigate drug metabolism. Human liver subcellular fractions, such as human liver microsomes (HLM) and S9 fractions, provide physiological relevance, experimental feasibility, and cost efficiency, making them preferred options for initial in vitro drug screening assays over purified enzymes [7,8,9]. HLM are derived from the endoplasmic reticulum membranes of liver cells and are vesicular fragments formed during cell homogenization and centrifugation. They are enriched with phase I enzymes, including various isoforms of cytochrome P450 (CYP), such as CYP3A4, CYP2C9, CYP2B6, and CYP2E1, which are primarily responsible for drug modifications like oxidation and hydroxylation. The S9 fractions, which contain a mixture of microsomes and cytosol, provide insights into both phase I and phase II metabolism.

**Scheme 1:**
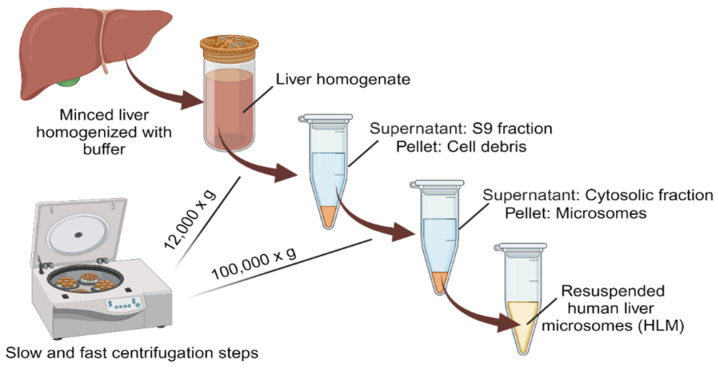
Preparation of various human liver subcellular fractions.

The isolation of HLM and S9 fractions is achieved through differential centrifugation of liver homogenates. Initially, low-speed centrifugation removes debris and nuclei, resulting in a supernatant that contains both cytosolic and microsomal components, collectively known as the S9 fraction. Subsequent ultracentrifugation of the S9 fraction isolates the microsomes, which form a pellet. This microsomal pellet is then resuspended in the buffer to obtain HLM [10]. A schematic illustration of this process is shown in Scheme 1.

The catalytic activity of CYP is influenced by its redox partner, cytochrome P450 reductase (CPR), which accepts electrons from nicotinamide adenine dinucleotide phosphate (NADPH). CPR contains cofactors of flavin adenine dinucleotide (FAD) and flavin mononucleotide (FMN), which facilitate electron shuttling from NADPH to the CYP heme center. This electron transfer process fuels the reduction of the CYP heme from Fe(III) to Fe(II), which then reacts with O_2_ to yield reactive Fe(IV) species that oxidize the substrate [11]. As an alternative approach, the above electron transfer process and the substrate oxidation can be facilitated by electrochemical means. This is achieved by immobilizing HLM or S9 films onto an electrode and activating them under an applied potential. This method falls into protein film electrochemistry, which is a powerful electroanalytical technique to study the properties of redox proteins and their catalytic features. By directly transferring electrons between the immobilized proteins and the working electrode, protein film electrochemistry allows for the visualization and quantification of redox processes happening within the protein without needing any natural redox mediators or cofactors [12].

Even though the direct electrochemistry of purified CPR and CYP is well understood [13,14,15,16,17], there are a handful of studies reported on activating such enzymes electrochemically in complex human liver subcellular fractions. Initial attempts of activation of CPR and CYP in liver microsomes were conducted by immobilizing them directly via physisorption or layer by layer via electrostatic interactions on graphitic and Au electrode surfaces [18,19,20]. Recently, the Krishnan group has reported direct electron transfer and electrocatalytic activity of such films on various electrodes [21,22,23], including nanostructured electrodes fabricated with carbon nanotubes [24,25], and magnetic nanoparticles [26]. They showed that the liver microsomal cytochrome P450 electrocatalysis could significantly improve the analytical sensitivity and metabolite production with appreciable film stability on the electrode.

Herein we report a comprehensive comparison of the direct electron transfer and electrocatalytic properties of HLM and S9 fractions when immobilized onto a high-purity graphite (HPG) electrode. Additionally, the electrochemically driven metabolite formation by S9 films was confirmed using high-performance liquid chromatography–mass spectrometry (HPLC-MS). Our findings highlight the similarities and differences in the electrocatalytic behavior of these HLM and S9 films, offering valuable insights for the development of novel biosensors, bioelectronics, and rapid, stable, one-step bioreactor designs for cost-effective electrochemical drug metabolism and inhibition assays.

## 2. Experimental

### 2.1. Materials and Reagents

The HLM and S9 fractions were purchased from XenoTech (now part of BioIVT, Westbury, NY, USA) and used as received. HLM has a total protein content of 20 mg/mL with a total CYP content of 0.579 nmol/mg and NADPH-cytochrome c reductase activity of 171 nmol/mg protein/min. The S9 fraction has a total protein content of 20 mg/mL with a total CYP content of 0.111 nmol/mg. Diclofenac sodium salt, phosphatidylcholine, and HPLC-grade solvents were purchased from VWR (Radnor, PA, USA). All reagents were of high-purity analytical grade (purity of ≥97%). All electrochemical measurements were carried out in phosphate buffer containing 0.10 M KCl, pH 7.0, at 25 °C. High-purity graphite rods (POCO EDM-4 grade) were purchased from McMaster-Carr (Atlanta, GA, USA) and cut into small cylinders (geometric electrode surface area = 0.2 cm^2^). PTFE electrode holders and metal shafts were purchased from Pine Research (Durham, NC, USA).

### 2.2. Microsomal and S9 Film Preparation

The HPG disk electrodes were polished with 120 grit size SiC paper, rinsed with Millipore water, and dried under nitrogen to obtain fresh surfaces for adsorbing HLM or S9. A film of HLM or S9 was formed on each freshly polished electrode by adsorbing from a 20.0 μL solution of the respective subcellular fraction for 30 min at 4 °C. The electrodes were rinsed with buffer to remove weakly bound and unbound material and then used for electrochemical studies.

### 2.3. Direct Electron Transfer Measurements

An electrochemical analyzer (CH Instruments, model 650E, Austin, TX, USA) was used for electrochemical experiments. The cyclic voltammetry (CV) measurements were made in a standard three-electrode cell consisting of an Ag/AgCl reference electrode, a Pt-wire counter electrode, and an HLM or S9-coated HPG working electrode. CVs were performed under anaerobic conditions by purging argon during the measurements. Square-wave voltammetry (SWV) was also used using the same electrode setup under similar experimental conditions to visualize the direct electron transfer features with minimized background charging currents.

### 2.4. Electrocatalytic Oxygen Reduction

The HLM and S9-catalyzed oxygen reduction was studied in stirred buffer solutions to achieve mass transfer by convection. The electrolyte buffer solution was saturated with oxygen by purging the solution 45 min prior to the experiment and during the experiment.

### 2.5. Electrocatalytic Diclofenac Hydroxylation and the Detection of Metabolites

The three electrodes adsorbed with S9 were placed in a stirred bulk electrocatalysis cell containing 100.0 μM Diclofenac in 10.0 mL of pH 7.0 potassium phosphate buffer. Electrolysis was carried out at an applied potential of −0.6 V vs. Ag/AgCl for 1 h under a constant supply of oxygen using a 8-channel electrochemical analyzer (CH Instruments, model 1040C, Austin, TX, USA). After the bulk electrocatalysis, the reaction mixture was analyzed by an HPLC-MS system (HPLC: Agilent, model 1260 Infinity, Santa Clara, CA, USA; mass spectrometer: Thermo Fisher Scientific, model LTQ-XL, linear ion-trap with heated electrospray ionization probe, Waltham, MA, USA). An Agilent ZORBAX RR Eclipse XDB-C8 column (2.1 × 100 mm, 3.5 µm) was used for all separation analyses. The LC parameters were as follows: injection volume, 10 µL; column temperature, 40 °C; mobile phases were 0.1% formic acid in water (A) and 0.1% formic acid in acetonitrile (B). The gradient started at 5% B and increased to 95% B over 7 min at a 0.4 mL/min flow rate, held at 95% B for 1 min, decreased to 5% B at 9 min, followed by a 1 min post-run at 5% B. Mass spectra were collected in three scan modes: a full scan from *m*/*z* 50–500, followed by two MS-MS-dependent scans (via collision-induced fragmentation) focusing on *m*/*z* ratios of 296 (diclofenac) and 312 (mono-hydroxy diclofenac). Then the extracted ion chromatograms and respective mass spectra were analyzed using Thermo Fisher Scientific X-calibur software (version 4.3).

### 2.6. Fourier Transform Infrared (FTIR) Spectroscopy

Liver microsomal and S9 films immobilized on the electrodes were characterized by FTIR spectroscopy (Thermo Fisher Scientific, model Nicolet IS50, Waltham, MA, USA) in the attenuated total reflectance (ATR) mode. The electrodes were mounted on an ATR diamond crystal, and 32 scans were acquired and averaged to obtain a good signal-to-noise ratio. The FTIR spectrum of the polished bare HPG electrode on an ATR diamond crystal was also obtained for comparison.

## 3. Results and Discussion

### 3.1. FTIR Characterization of HLM and S9 Films Immobilized on the HPG Electrodes

**Figure 1:**
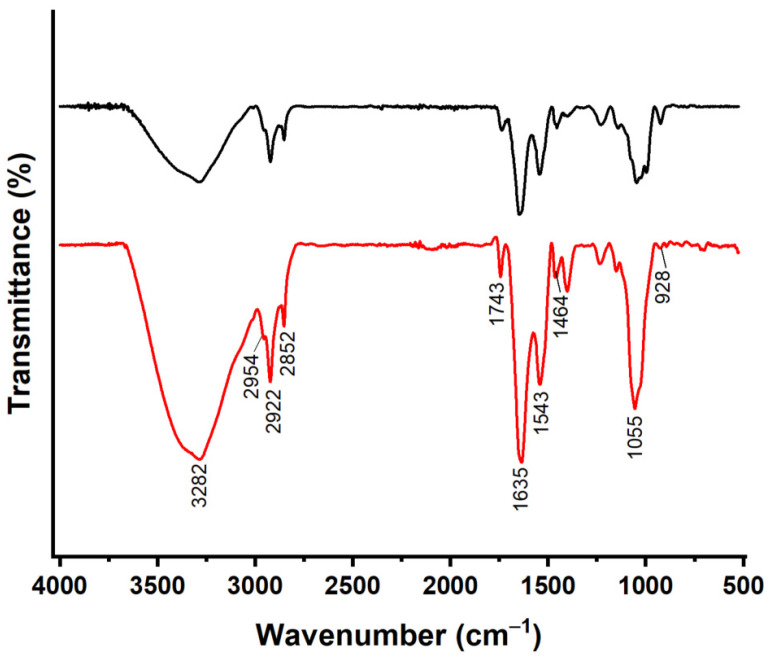
Representative FTIR spectra of HPG/S9 (red) and HPG/HLM (black) on an ATR diamond crystal.

The FTIR spectra for HPG/S9 and HPG/HLM films are shown in Figure 1. These spectra confirm the successful adsorption of HLM and S9 films onto the electrode surface, as evidenced by vibrational frequencies observed corresponding to membrane phospholipids and proteins. The peaks at 3282, 2954, 2922, and 2852 cm^−1^ correspond to the vibrational frequencies associated with –OH, –CH_3_ asymmetric vibrations, –CH_2_ asymmetric vibrations, and –CH_2_ symmetric vibrations in biomolecular hydrocarbon chains, respectively. Additional significant vibrational frequencies include the peak at 1743 cm^−1^, indicative of C=O ester stretching in phospholipids, and the peak at 1464 cm^−1^, characteristic of –CH_2_ bending vibrations in lipids [27]. The vibrational frequency range from 1300 to 900 cm^−1^ is characteristic of phosphate and choline groups commonly found in phospholipids. Moreover, the prominent peaks at 1236, 1151, 1055, and 928 cm^−1^ can be attributed to the vibrations of P=O, C–O, P–O, and C–N bonds, respectively [22,28].

The peaks at 1635 and 1543 cm^−1^ correspond to the amide I and amide II bands, which arise due to the protein components (including CPR and CYP) within the phospholipid structures [29,30]. These frequencies align with previous FTIR analyses of microsomal films, indicating the effective adsorption of the human liver S9 and HLM films on the electrode surface.

### 3.2. Electrochemical Probing of S9 Films and Electron Transfer Kinetics

In electrochemically driven reactions, electrons can be directly transferred to enzymes from the electrode instead of using natural electron donors like NADPH or NADP. Therefore, ensuring successful electron delivery from the electrode to the redox-active enzymes on S9 is crucial. To investigate this, we employed both CV and SWV electrochemical analysis techniques. In CV, the electrode potential is swept back and forth to observe oxidation and reduction processes, providing insights into the system’s electrochemical behavior. The SWV uses square wave pulses on a staircase potential, offering higher sensitivity and better reduction of background currents, making it effective for detecting less pronounced electrochemical systems. Here, the direct electron transfer properties of the S9 film attached to an HPG electrode were compared with those of similarly attached HLM films.

**Figure 2:**
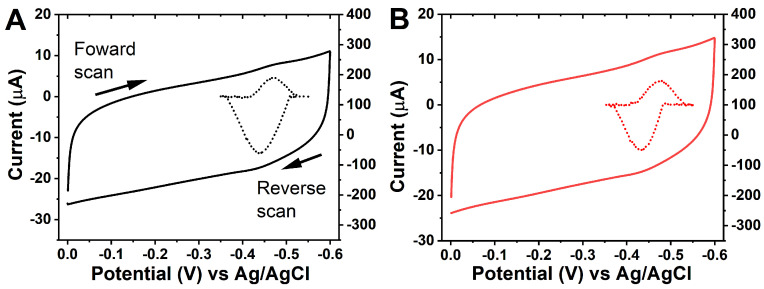
Cyclic voltammograms of (**A**) HPG/HLM and (**B**) HPG/S9 biofilms at scan rate 0.5 V s^−1^ in anaerobic (Ar-purged) phosphate buffer solution, pH 7.0. The solid line represents the data as acquired, while the broken line shows the background-subtracted data, eliminating the non-faradaic charging currents to enhance visualization of the redox peaks. The Y scale on the left corresponds to the background-subtracted data, while the Y scale on the right corresponds to the data as acquired.

Figure 2A,B presents the CVs of microsomal and S9 films adsorbed onto the HPG electrodes in a nitrogen atmosphere at pH 7.0. To enhance clarity, background capacitive current-subtracted cyclic voltammograms are also provided for both films (refer to the left Y-axis in Figure 2A,B). Both films demonstrated distinct redox features, indicating the activation of redox enzyme systems and their direct electron transfer with the electrode. To confirm that these redox features originated from the enzymes rather than the phospholipids, we used polished electrodes with an adsorbed control phospholipid film (L-α-phosphatidylcholine), which did not exhibit any redox peaks (Figure S1). The above results confirmed that direct electron transfer from the electrode to S9 enzymes was achieved, and it is comparable to HLM films.

Figure S2 shows plots of multiple cyclic voltammograms with varying scan rates for both HLM and S9 films. In both cases, the peak currents increased linearly with the scan rate. This linear relationship between the peak current and the scan rate confirmed the surface-confined redox chemistry in the microsomal and S9 films [31].

The formal potential (E°′) of the redox activity was estimated by calculating the average of the reduction and oxidation peak potentials. Observed formal potential values for both films were approximately −0.450 V vs. Ag/AgCl (Table 1), consistent with prior reports of the formal potential of the CPR enzyme [21,22,23,24,25,26,32,33]. The square wave voltammograms have also indicated that E°′ of the HLM and S9 biofilms were centered around −0.450 V vs. Ag/AgCl, and their peak current correlated well with the CV data (Figure S3). This similarity suggests that the direct electrochemistry of the human liver S9 fraction is analogous to that of HLM, where CPR enzymes actively accept electrons from the electrode. Moreover, previous studies indicated that the activation of CYP enzymes exhibited more positive potentials (−0.3 to −0.4 V vs. Ag/AgCl) [13,34,35,36], which rules out any involvement of CYPs in the direct electron transfer process based on our observations.

The peak width at half maximum (PWHM) is another parameter that indicates the stoichiometry of electrons (n) in the half-reaction. Ideally, at 25 °C, PWHM = 90 mV for n = 1 and PWHM = 45 mV for n = 2 [12]. The observed PWHM values for S9 and HLM films were 59 and 66 mV, respectively, aligning with previous reports for HLM immobilized on graphite electrodes [22,23]. The NADPH dependent cytochrome P450 oxidoreductase system (i.e., CPR), consists of FAD and FMN cofactors. These cofactors are essential for the electron transfer process from NADPH to cytochrome P450 enzymes, enabling them to carry out their catalytic functions, such as hydroxylation reactions. Prior studies indicated that FAD could accept up to two electrons from NADPH, whereas FMN acts as a one-electron carrier to reduce CYPs in the biocatalytic pathway [12,37,38]. It is important to understand that the PWHM value represents an average for all electron transfer processes between the electrode and the immobilized enzymes in the films. Therefore, based on our results and the literature, we suggest the possibility of mixed one- and two-electron transfer processes occurring in the direct electrochemistry of the designed microsomal and S9 films. This could be attributed to the orientation of CPR enzyme molecules embedded in the phospholipid bilayer and how they interact with the electrode surface. Overall, the examined electrochemical properties suggest non-ideal surface voltammetry of liver S9 and microsomal CPR enzymes.

The interfacial electron transfer rate constant (k_s_) for the heterogeneous electron transfer process between the HPG electrode and the CPR can be determined from plots of peak separation (ΔEp) and the logarithm of the scan rate using Laviron’s procedure [39]. This method relies on the exponential increase of the rate with overpotential, as derived from the Butler-Volmer equation [31]. The relationship between k_s_ and the peak separation for a molecule bound to an electrode surface is given by Equation (1).

k_s_ = (nFm × scan rate)/RT (1)

Here, n represents the number of electrons transferred, F is the Faraday constant (96,485 C mol^−1^), R is the universal gas constant (8.314 J mol^−1^ K^−1^), T denotes the temperature in Kelvin (298 K), and m correlates inversely with ΔEp in a non-linear relationship, based on Laviron’s derivation. Simply, the higher redox peak separation (or higher ΔEp) in the cyclic voltammogram indicates slower kinetics (which is a smaller k_s_) and vice versa.

**Figure 3:**
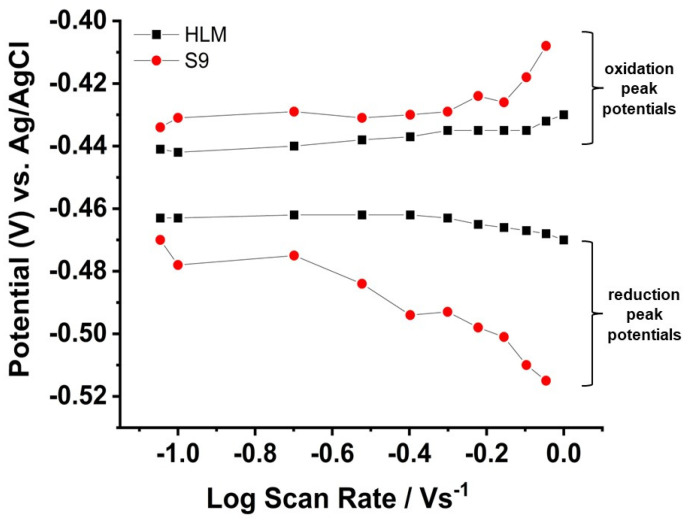
Trumpet plots displaying the oxidation and reduction peak potentials with logarithm of scan rate for HPG/HLM (black) and HPG/S9 (red) biofilms in anaerobic (Ar-purged) phosphate buffer solution, pH 7.0.

The peak separation between oxidation and reduction potentials with the logarithm of the scan rate is shown in Figure 3 for both HLM and S9 films. Our calculations indicated that the direct electron transfer rate of the HLM film is 4.7 times greater than that of the S9 film (Table 1), which is also evidenced by the peak separations shown in Figure 3. The slower electron transfer rate in the S9 can be attributed to the presence of electrochemically inactive phase II conjugation enzymes and other cellular components, which could hinder the kinetically favored electron transfer pathways. However, the S9 films exhibited a 1.25 times higher electroactive surface charge compared to the HLM film (Table 1). These findings suggest that, despite the presence of various enzyme types in the S9 fractions, including CYP, CPR, and conjugation enzymes, CPR interacts favorably with the electrode to facilitate the electron transfer process. This highlights the potential use of S9 fractions in electrochemical phase I drug metabolism assays similar to HLM.

### 3.3. Electrocatalytic Oxygen Reduction by the S9 Films

**Scheme 2:**
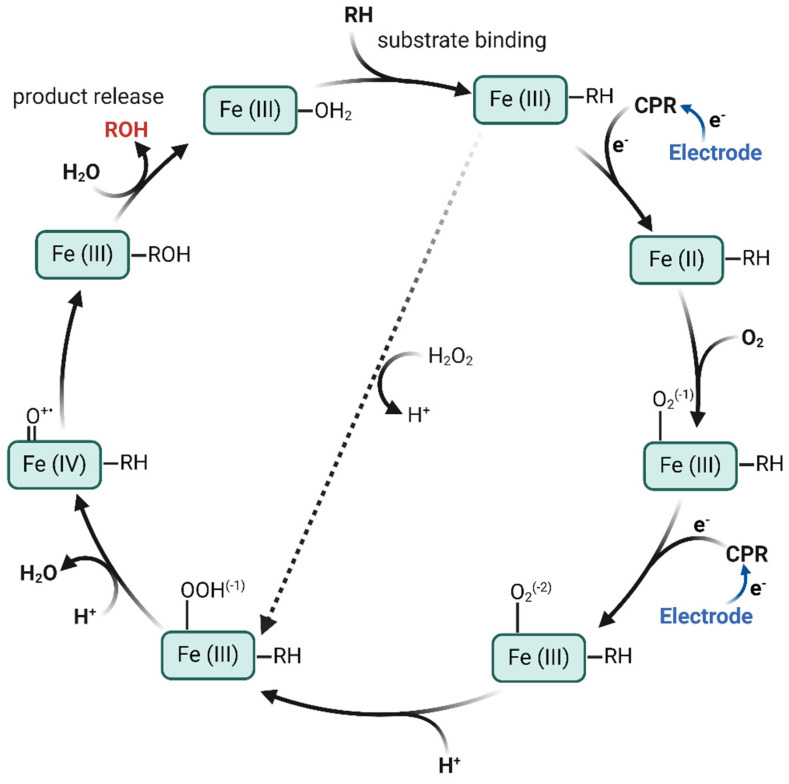
Proposed catalytic cycle of human CYP enzymes involving electrode as electron donor. The CYP catalytic cycle begins with the substrate (RH) entering the enzyme’s active site, displacing a water molecule, and forming an Fe(III)-RH complex. An electron from NADPH (in this case, from the electrode) reduces the iron heme center to Fe(II)-RH. Subsequent oxygen binding and a second electron transfer create an RH-Fe(III)-O_2_ complex. Protonation of this complex forms an RH-Fe(III)-OOH peroxo-complex, which then cleaves to produce a reactive [RH-Fe(IV)=O]^•+^ species. This oxo-iron species oxidizes the substrate to yield the hydroxylated product (ROH), returning the enzyme to its Fe(III) state and completing the cycle. Hydrogen peroxide can also facilitate this process by forming the RH-Fe(III)-OOH complex and following a similar pathway, known as the peroxide shunt pathway.

**Figure 4:**
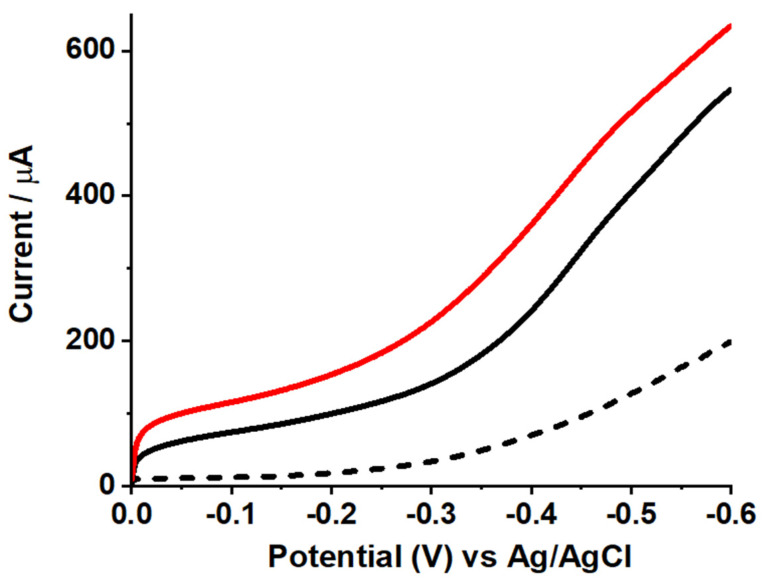
Cyclic voltammograms of HPG/HLM (solid black), HPG/S9 (solid red) and HPG/phospholipid (broken black) films at 0.5 V s^−1^ in stirred aerobic (O_2_-saturated) phosphate buffer solution, pH 7.0.

Oxygen is essential for cytochrome P450-catalyzed monooxygenase activity, to form the active CYP-heme oxidant, which subsequently transfers reactive oxygen to oxidize the bound substrate (Scheme 2). This catalytic pathway is initiated by electron mediation through CPR molecules from the electrode to the CYP heme centers, as evidenced by the electron transfer process described in the previous section. Under oxygen-rich conditions, even without the substrate, the heme-Fe(III) can be reduced to heme-Fe(II), which enables oxygen binding and its subsequent electrocatalytic reduction to peroxide, generating currents at the electrode [21]. Such electrocatalytic properties of the S9 film can be investigated through cyclic voltammetry experiments conducted under oxygen-saturated aerobic conditions [19,22,24]. We have observed enhanced steady-state current along with a positive shift in potential, which indicates the catalytic oxygen reduction reaction of CYPs in S9 and HLM films as shown in Figure 4. The electrocatalytic oxygen reduction currents from the immobilized S9 film are four times higher than those from the control phosphatidylcholine layer. For HLM films, this value is 3.2 times higher compared to the control film. This suggests that the electrocatalytic O_2_ reduction currents observed in HLM and S9 films are characteristic of CYP heme proteins.

### 3.4. Electrocatalytic Diclofenac Hydroxylation and Metabolite Identification Using LCMS Analysis

The proposed electrocatalytic cycle of CYP enzymes depicted in Scheme 2 can be described as follows. This cycle includes steps of substrate binding, electron transfer, and molecular oxygen activation, with the electrode serving as the primary electron donor [40,41]. Initially, the substrate (RH) enters the enzyme’s active site (i.e., the heme center), displacing a water molecule and interacting with heme Fe(III) to form an Fe(III)-RH complex. Following this, an electron transferred from NADPH via CPR reduces the iron to its ferrous state (Fe(II)-RH), which can also be achieved by the electrode without the need for NADPH, as shown in Scheme 2. Oxygen then binds to the Fe(II)-RH complex, creating a dioxygen adduct (RH-Fe(II)-O_2_). A subsequent electron transfer from CPR and protonation by the solvent results in the formation of a peroxo-complex (RH-Fe(III)-OOH). Then the cleavage of the O-O bond and release of a water molecule produce a reactive high-valent oxo-iron radical cation ([RH-Fe(IV)=O]^•+^). This oxo-iron species oxidizes the substrate by yielding the hydroxylated product (ROH), restoring the enzyme to its Fe(III) state. The hydroxylated product is then released, and a water molecule re-coordinates with Fe(III) heme center, completing the catalytic cycle. Additionally, hydrogen peroxide can act as an alternative electron and proton source, forming the RH-Fe(III)-OOH complex and following a similar pathway to generate the hydroxylated product (ROH). However, the efficiency of this peroxide shunt pathway is generally lower due to the limited H_2_O_2_ tolerance of most CYP450 enzymes.

Like in HLM, the S9 fraction contains CYP isotypes such as CYP3A4 and CYP2C9 [5]. These enzymes can convert the non-steroidal anti-inflammatory drug diclofenac into hydroxylated metabolites. In CYP bioassays, when exposed to HLM, diclofenac is primarily hydroxylated to 5′-OH-diclofenac by CYP3A4 and 4′-OH-diclofenac by CYP2C9 [42,43]. Prior electrochemical drug metabolic assays based on HLM have demonstrated similar results [21,26,44]. Therefore, we investigated the electrochemically driven drug metabolism of S9 films immobilized on HPG electrodes by monitoring the conversion of diclofenac to its hydroxy products. This was performed using bulk electrolysis in a stirred solution at a constant applied potential of −0.6 V vs. Ag/AgCl, followed by LC-MS detection of the metabolic products.

**Figure 5:**
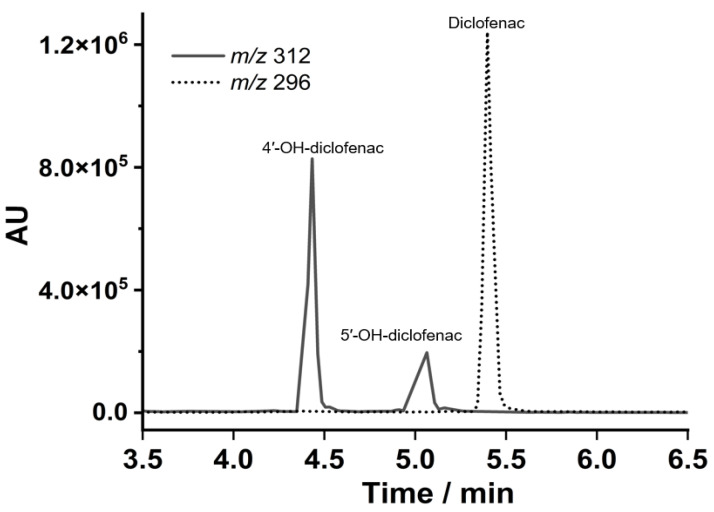
Extracted-ion chromatograms of the reaction mixture acquired for *m*/*z* 296 (broken line) and *m*/*z* 312 (solid line) after 1 h of electrolysis of 100.0 μM diclofenac solution in pH 7 phosphate buffer using HPG/S9 electrodes at −0.6 V vs. Ag/AgCl under a constant oxygen supply.

**Figure 6:**
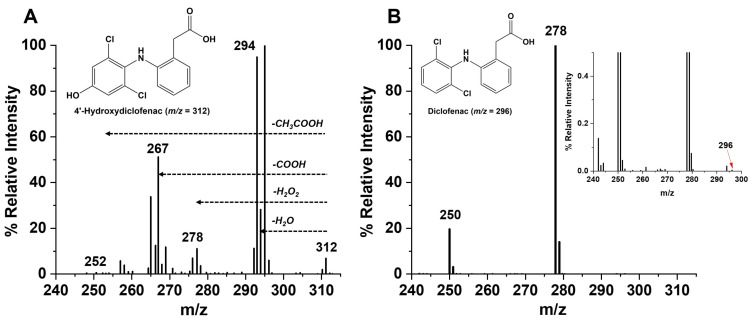
MS-MS spectra of the reaction mixture displaying mass peaks and fragmentation patterns for 4-hydroxydiclofenac (**A**) and diclofenac (**B**). The inset in (**B**) shows the lower intensity *m*/*z* 296 molecular ion peak of diclofenac. Bulk electrolysis was conducted using HPG/S9 electrodes at −0.6 V vs. Ag/AgCl, under a constant oxygen supply for 1 h in a 100.0 μM diclofenac solution prepared in pH 7 phosphate buffer.

As illustrated in Figure 4 and Figure 5, we detected both 4′-OH-diclofenac and 5′-OH-diclofenac metabolites. The extracted ion chromatogram at *m*/*z* 296 corresponds to the remaining diclofenac substrate in the assay mixture, while the extracted ion chromatogram at *m*/*z* 312 corresponds to the formed mono-hydroxy diclofenac products (Figure 5). The collision-induced dissociation spectra for *m*/*z* 296 (Figure 6A) and *m*/*z* 312 (Figure 6B) were also obtained to further confirm the structure of the metabolites. As depicted in Figure 6A, the *m*/*z* signals at 294, 278, 267, and 252 can be attributed to the loss of H_2_O, H_2_O_2_, COOH, and CH_3_-COOH from 4′-OH-diclofenac, respectively. Moreover, we confirmed that the polished bare HPG electrode with a phospholipid film in the absence of S9 did not catalyze the diclofenac hydroxylation under the applied potential of −0.6 V vs. Ag/AgCl (Figure S4). Interestingly, these observed results align with previous findings on the metabolic reactions of diclofenac catalyzed by HLM [21,44]. Therefore, we can confirm that the S9 films behave very similarly to HLM films when utilized in electrochemically driven drug metabolic assays.

## 4. Conclusions

In this study, we explored the feasibility of immobilizing human liver metabolic enzymes in the form of crude S9 fractions on an electrode surface to investigate the capability of performing electrochemically driven drug metabolic reactions. The advantage of such systems is to eliminate the need for traditional electron donors in the catalytic cycle. Additionally, enzyme co-factors or initial acceptors like CPR can directly receive electrons from the electrode, serving as an electron source for P450s. We demonstrated that this alternative electron transfer mechanism via electrodes to CYP systems can be achieved using a simple construction of a liver S9 fraction on an electrode, similar to our previous work with HLM [22]. Our observations confirmed the heterogeneous electron transfer process between the electrode and the CPR enzymes in the S9 film, as well as the electrocatalytic formation of drug metabolites by CYP enzymes through the diclofenac hydroxylation reaction.

We believe these simple bioelectrode designs incorporating liver subcellular fractions like S9 and HLM hold significant value for cost-effective drug metabolism and inhibition assays. Our future research will focus on enhancing the catalytic efficiency and stability of the S9 films by applying various nanostructure architectures to the electrodes. Furthermore, the integration of phase I and phase II enzyme systems in a combined electrochemical and chemical assay presents a promising approach for synthesizing and studying drug metabolites. Given the composition of liver S9 fractions, we envision developing a comprehensive assay that enables the synthesis of both phase I and phase II drug metabolites within a single assay system in the future.

## Figures and Tables

**Table 1:** Electrochemical parameters of HLM and S9 films adsorbed onto HPG electrode under nitrogen atmosphere.

Electrode Assembly	E°′/mV vs. Ag/AgCl	Q/nC	PWHM/mV	k_s_ (s^−1^)
HPG/HLM	−453 ± 3	275 ± 12	66 ± 1	66 ± 10
HPG/S9	−451 ± 1	344 ± 14	59 ± 2	14 ± 3

## Data Availability

The raw data supporting the conclusions of this article will be made available by the corresponding author on request.

## Supplementary Materials

The following supporting information can be downloaded at: https://www.mdpi.com/article/10.3390/metabo14080429/s1, Figure S1: CV of the control electrode; Figure S2: CVs with varying scan rates for both HLM and S9 films; Figure S3: Square wave voltammograms of HLM and S9 films; Figure S4: LCMS data for diclofenac standard solution.
