# Supplementary material for: Electrochemical Probing of Human Liver Subcellular S9 Fractions for Drug Metabolite Synthesis
Source: Metabolites. 2024 Aug 3;14(8):429. doi: 10.3390/metabo14080429 (PMC11356027; doi:10.3390/metabo14080429)
Supplement: Supplementary file 1 [file metabolites-14-00429-s001.zip › metabolites-3130293-supplementary.pdf]

Supplementary Material

Electrochemical Probing of Human Liver Subcellular S9 Fractions for Drug Metabolite Synthesis

Daphne Medina, Bhavana Omanakuttan, Ricky Nguyen, Eman Alwarsh and Charuksha Walgama *

Department of Physical & Applied Sciences, University of Houston-Clear Lake, 2700 Bay Area Boulevard, Houston, TX 77058, USA

* Correspondence: walgama@uhcl.edu; Tel.: +1-281-283-3785

Table of Contents

S-1: Cyclic voltammogram of the control electrode

S-2: Cyclic voltammograms with varying scan rates for both HLM and S9 films

S-3: Square-wave voltammograms of HLM and S9 films

S-4: LCMS data for diclofenac standard solution

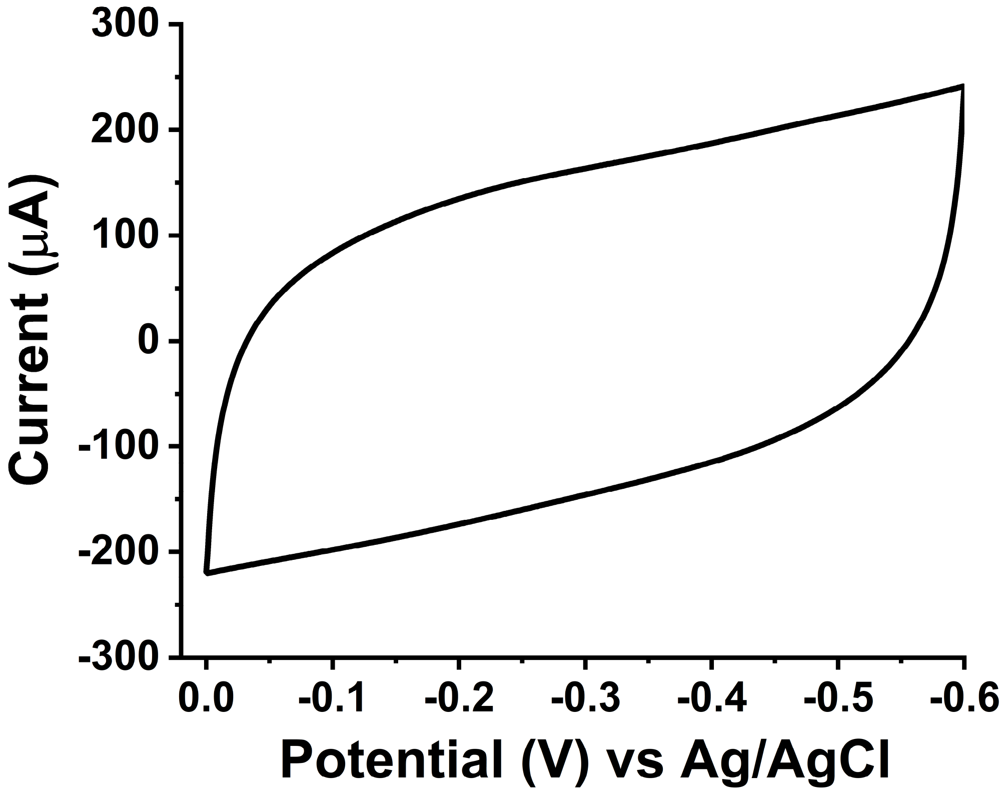

Figure S1. Cyclic voltammograms of control phosphatidylchloine film adsorbed onto HPG electrode at 0.5 V s^{-1} in anaerobic (Ar purged) phosphate buffer solution, pH 7.0.

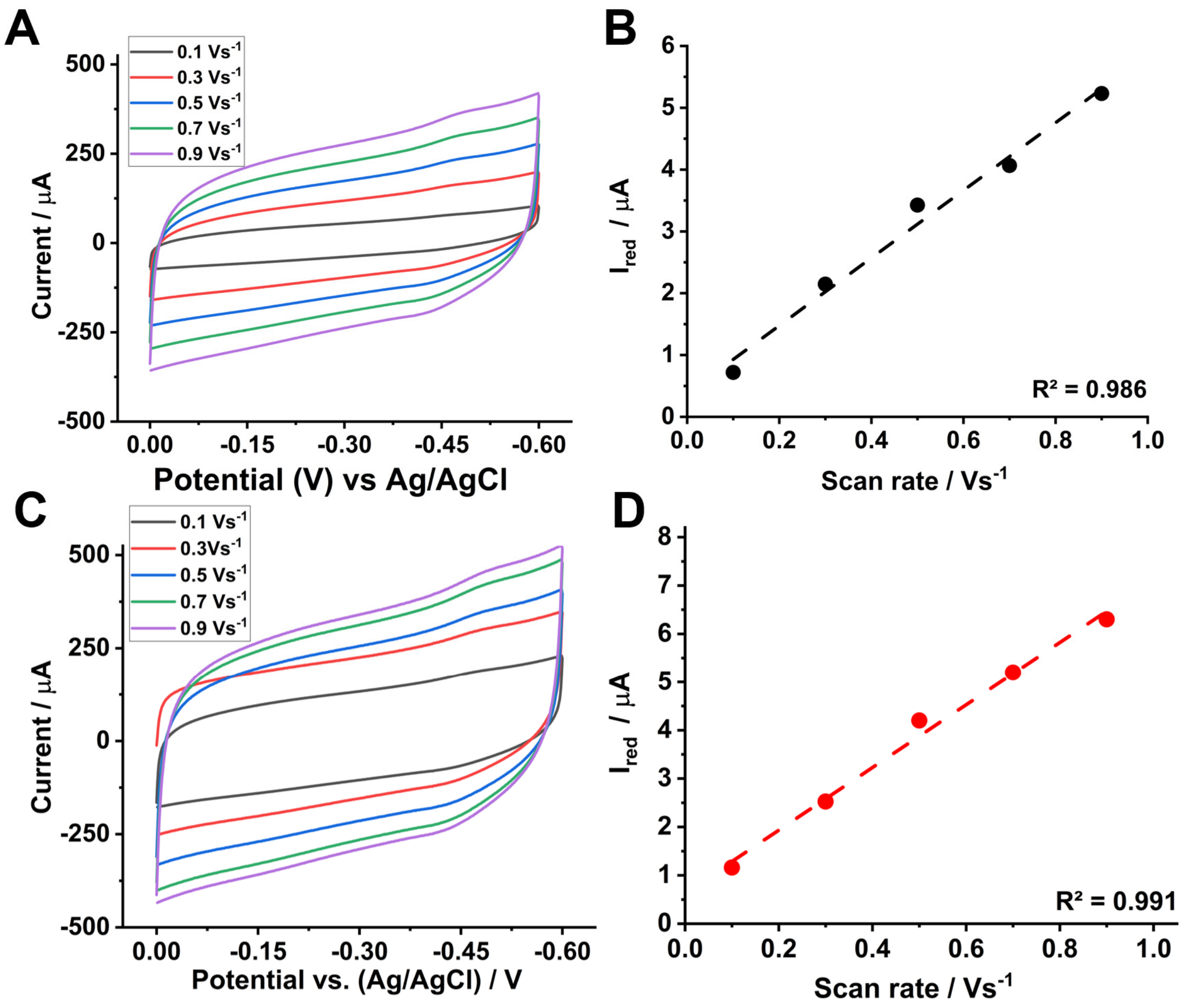

Figure S2. Representative cyclic voltammograms and corresponding scan rate vs. current plots for the prepared HPG/HLM (**A, B**) and HPG/S9 (**C, D**) films. Cyclic voltammograms were conducted at scan rates of 0.1-0.9 V s–1 in pH 7.0 phosphate buffer.

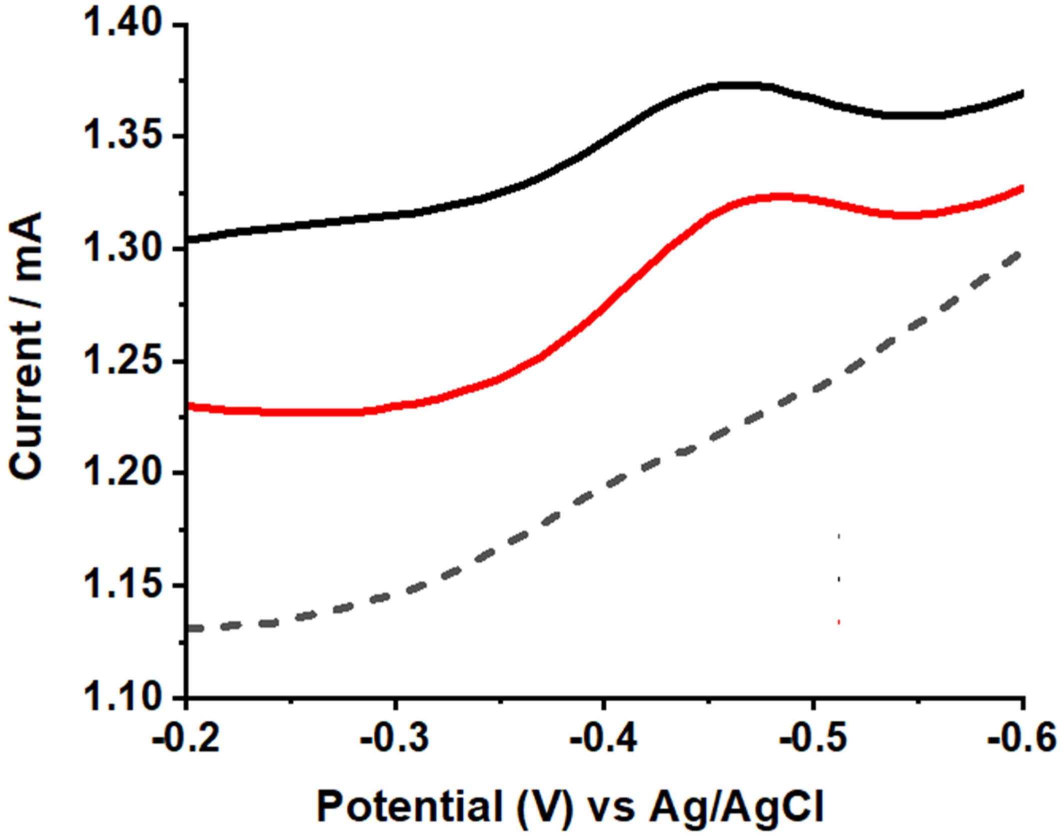

Figure S3. Square-wave voltammograms of HLM (black), S9 (red) and phosphatidylchloine(dashed) films adsorbed onto HPG electrodes. Amplitude 90 mV, frequency 20 Hz, voltage increment 10 mV in anaerobic (Ar purged) phosphate buffer solution, pH 7.0.

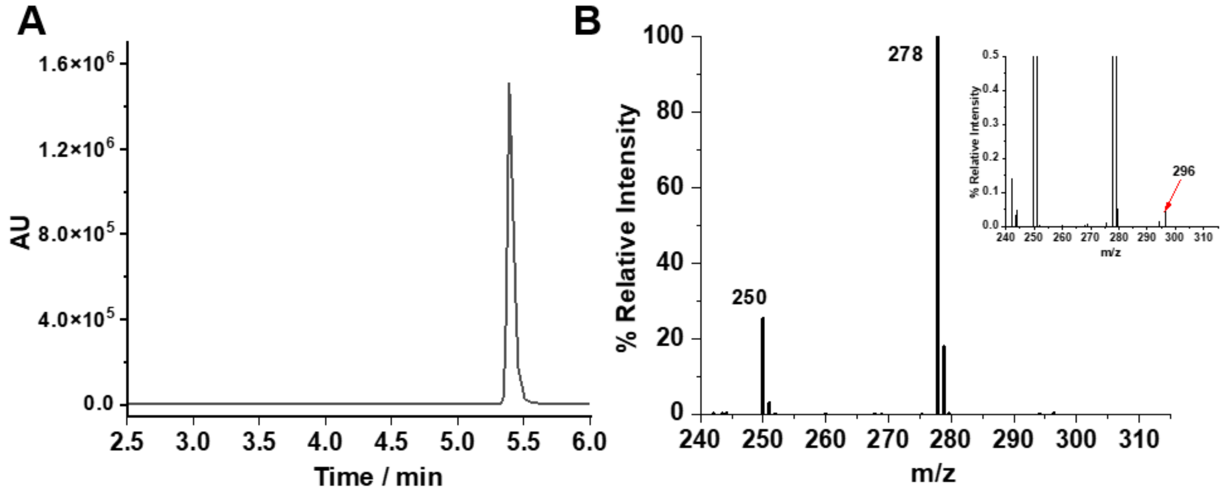

Figure S4. Extracted-ion chromatogram (**A**) and MS-MS spectrum (**B**) of the reaction mixture acquired for m/z 296 after 1 h of electrolysis of 100.0 μM diclofenac solution in pH 7 phosphate buffer using HPG/phospholipid control electrodes at −0.6 V vs Ag/AgCl under a constant oxygen supply.
